# Enhancing the Self: Amateur Bodybuilders Making Sense of Experiences With Appearance and Performance-Enhancing Drugs

**DOI:** 10.3389/fpsyg.2021.648467

**Published:** 2021-06-11

**Authors:** Juraj Macho, Jiri Mudrak, Pavel Slepicka

**Affiliations:** Department of Pedagogy, Psychology and Didactics, Faculty of Physical Education and Sport, Charles University, Prague, Czechia

**Keywords:** doping, self, self-concept, sense making, interpretative phenomenological analysis, appearance and performance-enhancing drugs, anabolic steroids, bodybuilding

## Abstract

In this paper, we implemented a methodological framework of interpretative phenomenological analysis (IPA) and a theoretical conceptualization of multiple selves to explore the subjective accounts of six amateur bodybuilders using appearance- and performance-enhancing drugs (APEDs). The participants made sense of their bodybuilding careers and experiences with APEDs in a way that showed a multiplicity and complexity of reasons for using APEDs, which stemmed from tensions they perceived between the context of bodybuilding and other life domains. The participants’ reasons for the use of APEDs included not only enhancing their body, appearance and performance but also enhancing other subjectively important psychological characteristics, such as agency and self-control, the development of knowledge and expertise, sense of meaning, well-being, and quality of life. In the analysis, we integrated these themes through the concept of the “extraordinary self,” based on which our participants strived for self-actualization through bodybuilding and the use of APEDs. In the sense making of our participants, a potential “exit point” subverting their APED use emerged from a tension between such “extraordinary selves” and the “ordinary selves” through which they perceived APEDs as preventing them from living normal, balanced lives outside the context of bodybuilding. However, success in balancing the two selves also created the possibility of the future use of APEDs.

## Introduction

The evolution of modern sports through hegemonic ideals of accomplishment has been intimately intertwined with the use of performance-enhancing substances (Dimeo, 2007). For a long time, doping was exclusively considered to be a problem of elite sports, but appearance- and performance-enhancing drugs (APEDs) gradually found their way to leisure sports, including amateur bodybuilding and fitness, and permeated a broad population of regular clients of gyms, fitness centers, and the general fitness community (Backhouse et al., 2007; Sagoe et al., 2014; McVeigh et al., 2015). Gyms and fitness centers have been found to represent the type of environment in which individuals are the most susceptible to doping, especially the use of anabolic steroids (Simon et al., 2006; Striegel et al., 2006). Questionnaire studies have suggested that there is a great variability in the prevalence of APEDs in regular gym goers ranging from 3,9% in Sweden (Leifman et al., 2011), 5% in the United States (Kanayama et al., 2001), 11,1% in Portugal (Tavares et al., 2020), 11,6% in Cyprus (Kartakoullis et al., 2008), 13,5% in Germany (Striegel et al., 2006), and up to 22% in the United Arab Emirates (Al-Falasi et al., 2008). In the United Kingdom context, Grace et al. (2001) found the prevalence of doping among members of gyms popular among bodybuilders to be as high as 53%. Bodybuilding also represents the sport with the most frequent use of doping (22%) among all sports tested by the WADA (World Anti-Doping Agency, 2019).

In the bodybuilding context, APEDs were originally used mainly by competitive bodybuilders (Lenehan et al., 1996). However, presently, we can see a much more diverse picture regarding both the characteristics of APED users and their motivations (McVeigh et al., 2015; Christiansen et al., 2017). Quantitative research studies focusing on doping in sports highlight two main motivations for doping: self-realization through performance, especially in professional sports, and self-realization through appearance, especially in amateur sports (Ehrnborg and Rosen, 2009). Current theoretical frameworks of the motivation for doping have proposed the hypothesis that the reason *“why”* a person engages in sports may affect “*how”* that person behaves in sports and the means he or she uses to achieve his or her goals (Vallerand and Losier, 1994) including doping. In this context, researchers have often used an integrated approach that combines self-determination theory (SDT; Ryan and Deci, 2000), achievement goal theory (AGT; Nicholls, 1984) and planned behavior theory (Chan et al., 2015). According to this approach, doping is considered an intentional behavior that is to a certain degree determined by attitudes and beliefs about doping (Mudrak et al., 2018). In addition, doping has been described as a result of unfulfilled basic psychological needs for autonomy, competence and relatedness (Ryan and Deci, 2000; Donahue et al., 2006; Barkoukis et al., 2011; Hodge et al., 2013; Mudrak et al., 2018); a dominant ego goal orientation (Sas-Nowosielski, 2006; Sas-Nowosielski and Swiatkowska, 2008; Barkoukis et al., 2013; Allen et al., 2015) or subjective doping norms (Chan et al., 2015).

However, the qualitative doping research focusing specifically on the knowledge, attitudes, beliefs, motives and experiences of bodybuilders and recreational gym goers using APEDs offers deeper insight into their traits, characteristics, and motivations and into the mutual interconnections between individual and social factors (Grogan et al., 2006; Petrocelli et al., 2008; Bjørnestad et al., 2014; Kimergård, 2015; Van Hout and Kean, 2015; Greenway and Price, 2018). In an evidence-based socioecological framework, Bates et al. (2019) point out the complexity of APED use and identify an interaction among a range of factors at the individual, social network, institutional, community, and societal levels. In a similar vein, Petróczi and Aidman (2008) approach APED use through the life-cycle model of performance enhancement in which they understand APED use as resulting from interactions among motivational, systemic and situational factors that constitute numerous “entry” and “exit” points throughout the athletes’ development. Based on their own interviews and a systematic review of the current research, Christiansen et al. (2017) developed a unified framework in which they strove to reduce the complexity of motivations for APED use in fitness and strength training environments. They proposed a typology of APED users including four ideal types: (1) the expert type who is motivated by lay scientific curiosity and fascination with pharmacological performance enhancement; (2) the well-being type who is motivated by vanity and/or wishes for restoration or rejuvenation; (3) the athlete type who is motivated by competitive aspirations; and (4) the YOLO (“you only live once”) type who is motivated by impatience, curiosity and influence from peers and authoritative role models.

One of the most influential ethnographic studies offering detailed insight into bodybuilding culture (Klein, 1993) explains bodybuilding in terms of gender inadequacy caused by a masculinity-in-crisis. Bodybuilding is conceptualized as *“at the very least, a subculture, whose practitioners suffer from large doses of insecurity”* (Klein, 1993, p. 174), and muscular bodies are, according to Klein, a *“psychologically defensive construct that looks invulnerable but truly only compensates for self-perceived weakness”* (Klein, 1993, p. 18). Klein’s conclusions about bodybuilders’ insecurity and preoccupation with appearance have contributed to a worsening reputation of bodybuilding among the wider public, and psychiatrists have begun to describe a new category of young patients obsessed by their body and muscles (Andreasson and Johansson, 2019). Academics have increasingly considered bodybuilding from a pathologizing perspective in which bodybuilders are associated with obsessive perfectionism, anhedonia and pathological narcissism (Davis and Scott-Robertson, 2000); dissatisfaction with one’s body; a body image disorder referred to as *“muscular dysmorphia”* or “bigorexia” (Pope et al., 1993; Peters and Phelps, 2001; Skemp et al., 2013); obsessive-compulsive disorder (Pope et al., 2000); or a risk of developing exercise addiction (Berczik et al., 2012). There are also studies describing the psychological and behavioral similarities between bodybuilders and anorexia nervosa and bulimia nervosa patients characterized by an elevated likelihood of developing an eating disorder (Goldfield et al., 2007).

Based on his qualitative research, British sociologist Lee F. Monaghan to a certain degree opposes this pathologizing perspective and the findings presented by Klein (1993). Monaghan (2001b) argues that hegemonic masculinity is not the only meaning that may be ascribed to a muscular body. He considers theories that explain bodybuilding and APED use through the prism of gender inadequacies and insecurities caused by “masculinity-in-crisis” as too narrow and insufficient. He explains the consumption of APEDs and other risky bodybuilding practices as to a certain degree a form of embodied pleasure, the sensual bodily pleasures of anaerobic exercise and the perception of apparent psychosocial benefits: *“The ‘steroid pump’, as it is known in gym argot, is an extremely intense experience treated with almost mystical reverence”* (Monaghan, 2001b, p. 348). Monaghan also highlights the importance of the social processes that influence bodybuilders in defining the type of “muscular body” toward which they orient themselves. In this way, hard-core bodybuilders engage in a “social process of becoming” (Monaghan, 2001b, p. 83) during which they learn to recognize “sizably muscular and exceptionally lean bodybuilding physiques” as aesthetically pleasing and develop a perspective on muscular bodies that is different from that of people who do not participate in the bodybuilding subculture.

Christiansen and Flegal (2020) argues that while Klein’s approach involves a risk of drawing a stereotypical picture of bodybuilders as a homogenous group of psychologically vulnerable men, Monaghan’s approach, inspired by Giddens’ concept of a plastic identity, involves the risk of atomizing our understanding of the motivations for the use of APEDs. In his book focused on gym culture, identity and APEDs, Christiansen explains APED use from the perspective of evolutionary psychology, more specifically from the perspective of the theory of precarious manhood and from the perspective of identity construction. Rather than a social construct, Christiansen understands bodybuilders’ identity as something that is plastic, mutable and constantly up for renegotiation. From this perspective, bodybuilders must find and continuously work on their own identity, which has to be confirmed and validated by their significant others. Bodybuilders’ body image and hence their identity are more than a social construction as these subjective constructions are bound to the real, physical world and develop in the bodybuilders’ interactions with the real world. According to Christiansen, such interconnectedness between body image, identity and objective reality may explain why some bodybuilders strive to accelerate the pace of their body-altering efforts by supplementing their physical training with steroids.

In addition to Andreasson and Johansson (2020) and Christiansen and Flegal (2020) highlight the concept of identity as one of the central points in their recent qualitative study dealing with the trajectories and processes of becoming and unbecoming a fitness doping user. The authors describe identity as tightly interwoven with the concepts of trajectories and learning processes and they show how fitness doping is negotiated in relation to gender, health, individual freedom and doping-related policies. For example, in the Swedish context, APED users may experience a threat to their identity due to a potential risk of being perceived as deviant because APED use is illegal here. As one participant in Andreasson and Johansson (2020, p. 105) study states, *“Someone may knock on your door at any time. You could get dragged out of your bed when sleeping, even if you’re a regular person, and all you’re doing is going to work*… *Unfortunately, Sweden’s legislation is such that you’re not allowed to use steroids, and if you do, you’re considered a criminal. Even if you aren’t, the law says you are.”* In other contexts, such as the Czech Republic, in which the personal use of APEDs is not prohibited, such identity negotiation may be significantly different, shaping the ways in which APED use is approached, initiated, and experienced.

### Theoretical Framework: Multiple Selves and Sense Making

The theoretical frameworks, models and typologies introduced above recognize a wide range of factors influencing APED use in the fitness and bodybuilding contexts including personal, systemic, institutional, and policy levels. However, we argue that they do not sufficiently consider a phenomenological perspective and the ways in which APED users make sense of their experience with APEDs in the context of their bodybuilding careers as well as other life domains. Although the above-introduced frameworks represent valuable heuristic tools that deepen our understanding and greatly contribute to exploring APED use from multiple angles, the explanation of this complex intentional behavior needs to take more into account the subjective perceptions of individual APED users, which have been relatively neglected. A notable exception represents Andreasson and Johansson’s (2020) qualitative study considering the subjective perspectives of APED users on their trajectories of becoming and unbecoming doping users, which we aim to develop further in our study. Hence, our main goal is to explore how individuals make sense of APEDs and their APED experiences, how they perceive their own agency in relation to the use of APEDs and how they relate to other subjectively important contexts that shape their decision to use or stop using APEDs.

The framework that we use in our study stems from the concept of the self, i.e., “an organized knowledge structure representing people’s own individual understandings of themselves,” which is partly abstract and partly autobiographical (Kihlstrom and Kihlstrom, 1999, p. 23). In this context, it is important to distinguish between the concept of the self as an individual phenomenon derived predominantly from experience and the concept of identity, which focuses on socially constructed aspects of individual self-understanding (Sharma and Sharma, 2010). We can illustrate this distinction in the bodybuilding context by contrasting an analytical emphasis on the experiential process of becoming a doping user versus approaching APED use through the prism of hegemonic masculinity (Andreasson and Johansson, 2020). In this way, we focus predominantly on the self as an experiential phenomenon in our study, which is “central to the sense-making process and serves as a useful concept for exploring the interrelationship between individuals, their bodies, larger relational, social and cultural systems and between the private and the public domains of our own experience” (Smith and Osborn, 2007, p. 519).

According to Markus and Wurf (1987), the self-concept is a dynamic interpretative structure that mediates most significant intrapersonal processes and a wide variety of interpersonal processes. The working self-concept is a particular configuration of representations drawn from the self-concept that regulates individuals’ ongoing actions and reactions. Thus, individuals’ behavior is regulated according to whatever set of dynamic structures (such as possible selves or self-schemas) are currently activated in the working self-concept. The influence of the working self-concept in the shaping and controlling of behavior can be seen in two classes of behaviors. Intrapersonal processes, which include self-relevant information processing, affect regulation and motivational processes, and interpersonal processes, which include social perception, social comparison, and seeking out and shaping interaction with others. People both shape and are shaped by their social interactions. The self-concept provides a framework that guides the interpretation of one’s social experiences but that also regulates participation in these experiences.

One of the dynamic self-structures or types of self-knowledge is the concept of possible selves (Markus and Nurius, 1986). Possible selves derive from representations of the self in the past; they include representations of the self in the future and individuals’ ideas of what they might become, what they would like to become, and what they are afraid of becoming. The possible selves are important because they function as incentives for future behavior – they are selves to be approached or avoided but also provide an evaluative and interpretive context for the current view of self. They are different and separable from the current self but are intimately connected to it. The possible selves are individualized or personalized, but they are also distinctly social. Many possible selves are the direct result of previous social comparisons in which the individual’s own thoughts, feelings, characteristics and behaviors have been contrasted to those of significant others. The pool of possible selves derives from the categories made salient by the individual’s particular sociocultural and historical context and from the models, images, and symbols provided by the media and by the individual’s immediate social experiences.

## Methodology

### Design of the Study

Therefore, we approached the APED use of amateur bodybuilders participating in our study from the perspective of qualitative psychology, and we analyzed the ways in which they made sense of their experiences with APEDs in the context of their multiple selves including their self-schemas of who they are, who they have been, and who they can be and want to become, both in and outside bodybuilding. The main research question was “How do amateur bodybuilders with APED experience make sense of their APED use inside and outside the context of bodybuilding?” Methodologically, we based our research on interpretative phenomenological analysis (IPA, Smith et al., 1999; Larkin et al., 2006; Smith and Shinebourne, 2012). The focus of IPA is subjective experience, as the method is designed to “explore in detail how participants are making sense of their personal and social world” (Smith and Osborn, 2008, p. 53). Another important aspect of IPA is that it is interpretative, i.e., it recognizes the active role of the researcher in the process of analysis. IPA involves a two-stage interpretation process whereby the participants make sense of their lived world and the researchers seek to make sense of the participants’ sense making. In this way, researchers not only describe participants’ sense making but also ask critical questions that allow them to understand some of the underlying meanings of which the participants may be less aware (Smith and Osborn, 2008). IPA is also idiographic; i.e., it emphasizes the close examination of individual case studies rather than the broader analysis of large populations (Smith and Osborn, 2008). Finally, the concept of the self is frequently used as an interpretative framework in IPA studies (Smith and Osborn, 2007; Shinebourne and Smith, 2009; Smith, 2019) as it allows for integrative explanations of the diverse and sometimes contradictory sense making of the participants.

### Participants

Six amateur bodybuilders with APED experience participated in our study, which corresponds with the IPA recommendations (Smith and Osborn, 2008). By including relatively few participants, we were able to conduct an in-depth examination of their experience and develop an analytic generalization of our findings toward the concept of multiple selves, which facilitates an understanding of APED sense making even beyond the experience of our participants (Yin, 2013). All participants lived in the Czech Republic with an exception of Daniel, who recently resided to the United States. We provide details about the participants’ bodybuilding careers and APED experience in Table 1. To obtain a homogenous sample, we included only men between 20 and 30 years old with at least several months’ experience using APEDs. Therefore, the participants were recruited by purposeful sampling in which two of the participants were acquaintances of the first author and the rest of the participants were recruited by a “snowball method,” i.e., they were recommended by participants who were already engaged in the study. To ensure participant anonymity, we refer to them using the pseudonyms Karel, Martin, Petr, Denis, Robert, and Daniel. The study was approved by the ethics committee of the Faculty of Physical Education and Sport of Charles University and was conducted in accordance with research ethics principles, including obtaining the informed consent of the participants and ensuring participant anonymity.

**TABLE 1:** Characteristics of the participants.

**Name**	**Age**	**Bodybuilding competitions**	**No. of cycles**	**Longest cycle**	**Substance used**
Karel	29	Yes (N, I)	1	1,278 days – blasting and cruising method^1^	Testosterone, boldenone, trenbolone, stanozolol, mesterolone
Martin	30	Yes (N)	8	9 months	Testosterone, nandrolone, metandienone, oxymetholone, stanozolol, oxandrolone, trenbolone, drostanolone
Petr	23	No	1	4 months	Testosterone, boldenone, stanozolol
Denis	24	Yes (N, I)	8	12 months	Testosterone, metandienone, nandrolone, stanozolol, drostanolone, boldenone, trenbolone, clenbuterol, oxandrolone, insulin, growth hormone
Robert	21	No	5	4 and 5 months	Testosterone, nandrolone, boldenone, metandienone, oxymetholone, trenbolone
Daniel	30	Yes (N, I, natural competitions)	1	Testosterone Replacement Therapy	Testosterone

*N, national competition; I, international competition; ‘Blasting’ and ‘Cruising’ Method is a way of using AAS in which using of higher doses of AAS for approximately 6 – 12 weeks (blast) are alternated by lower doses of AAS (cruise) for a similar length of time before starting another blast (Alcohol and Drug Support, 2021).*

Karel (29 years) has participated in several national and international bodybuilding competitions over the last 4 years, and at the time of the interview, he put his competitive career on hold and was reconsidering his future direction in bodybuilding due to his negative experience with APEDs.

Martin (30 years) has participated in five bodybuilding competitions. At the time of the interview, he had stopped competing and using APEDs, as his focus was on starting a family. However, he did not rule out a future return to competition and the use of APEDs.

Petr (23 years) went through a 4-month anabolic cycle consisting of a volume phase and a definition phase. Petr did not compete in bodybuilding, and based on his conflicting APED experience, he did not plan to continue using APEDs although he did not entirely rule it out in the future.

Denis (24 years) has participated in Czech and international bodybuilding competitions. He completed multiple anabolic cycles and had the most extensive APED experience of all the participants including the use of a wide range of anabolic steroids, insulin and growth hormone.

Robert (21 years) did not participate in bodybuilding competitions although he reported extensive experimentation with APEDs and did not rule out that he would continue using APEDs and possibly compete in the future.

Daniel (30 years) participated in two natural bodybuilding competitions at the beginning of his bodybuilding career. Then, he moved to the United States to become a model. At that time, he developed an eating disorder and went through multiple periods of depression. In the past year, he has returned to fitness and has begun injecting testosterone.

### Data Collection

The data were collected through semistructured interviews including themes related to the development of participants’ bodybuilding careers, their experiences with APEDs and fitness/bodybuilding lifestyles and their influence on other aspects of their lives. The interview was divided into seven sections – athletic beginnings, entry into the gym, bodybuilding competitions, APED experience, end of APED use, fitness lifestyle and potential future return to using these substances. First, the questions focused on general topics (e.g., “What was the first sport in which you participated?”, “Why did you start working out?”, “What motivated you to participate in bodybuilding competition?”, “Describe your experience with APEDs,” What positive/negative effects did you perceive?”, and “Why did you stop using these substances?”) followed by additional questions to uncover further details. Although all interviews opened with the same initial question, the participants’ answers affected the sequences and content of the follow-up questions. All participants were very involved in the interviews, and they frequently provided further insights without being prompted by the interviewer. After the interview, most of the participants reflected positively on the experience and even expressed gratitude for being involved in an interview in which they could openly describe their experience without feeling threatened or judged. The interviews took place in a secluded private space at a fitness center and in one case online. Each interview lasted approximately 60–90 min.

### Data Analysis

The interviews were recorded and later transcribed verbatim. The transcripts used the participants’ pseudonyms and omitted information that might allow for the identification of the participants. The interview transcripts were then analyzed following IPA recommendations (Smith and Shinebourne, 2012). The analysis was performed using the Atlas.ti software. First, we familiarized ourselves with all the interviews by repeated reading and rereading. After that, we went through all the interviews, starting with Karel, and we coded all the relevant statements that we developed further into more general categories. These categories were then gradually linked into broader themes that were in turn clustered into the main theme of each interview. Finally, we linked all the individual themes to superordinate themes that connected the experiences of all the participants. In the next section, we first present the results of our analysis of individual cases, which underscores the diversity of the participants’ sense making about their APED experiences. Then, we present the results of our analysis of the superordinate themes that illustrate the ways in which the participants made sense of their APED experiences in the context of multiple selves related to their bodybuilding careers as well as their lives outside bodybuilding.

## Results

### Amateur Bodybuilders Making Sense of APEDs

The participants described their bodybuilding careers as individualized self-projects in which they construed themselves in different ways and followed different goals that shaped their sense making about APED use. Through such sense-making, the participants related their bodybuilding and APED experiences to diverse themes including body, agency, control, excellence, expertise, health, relationships and well-being. However, in each case, a dominant theme was related to “becoming,” as the participants perceived that they enhanced their selves through bodybuilding and the use of APEDs in ways that were unique and out-of-the-ordinary. In some cases, this was complemented by themes related to “losing” as the participants perceived that by becoming extraordinary through bodybuilding, they forfeited a chance to live ordinary, “normal” lives.

#### Karel and Daniel: Becoming Bodies

Karel and Daniel both commented that throughout their sports careers, they strived to eliminate their unwanted selves, including an unattractive physique and a lack of agency, by developing excessive self-control of their bodies. As they elaborated further, this excessive self-control gradually shifted to a loss of control as their bodies became the dominant parts of their self-concept with detrimental effects on other subjectively important life domains. Metaphorically, they described themselves as gradually “becoming their bodies” and “losing their other selves” in the process. Interestingly, they made sense of their APED use in contrasting ways, which reflected how they perceived the effects of APEDs in this process of becoming and losing.

Karel described that he strived to eliminate the unwanted fat body and “*meaningless party life*” by developing his body through strict self-control. He spoke of a lack of agency during his early sports career, which he related to his inexperience and lack of self-regulation skills, which he also associated with having received misleading information from other people. In this context, Karel perceived his early training methods as “*complete non-sense”* or *“absolutely crazy*,” but he gradually overcame these obstacles and developed his interest in fitness as well as his agency. Karel: *“Eventually, I got so hooked on fitness, and I enjoyed fitness so much, that I could see myself in it and realized how important nutrition was as part of fitness, how important training was and, as a third pillar, how important regeneration was.”* However, with this growing interest in bodybuilding, including competitive ambitions, he developed a regimen of self-control to such an excessive level that it began to dominate his life. In this context, he described using APEDs and other substances, such as sleeping pills and light drugs, as a means of enacting even stricter control over his body, which had resisted some of his previous controlling attempts. Karel: *“Getting ready for competition started to go too far with an extreme focus exclusively on the body and bodybuilding. From the start, I set up a regime where I went to the gym in the morning and then went to work. My life revolved around precise calorie counts, there was not a single day when I would not know exactly how many calories I consumed. [*…*] My weight was good and I was in a good shape. But I refused to see what the cost of this was. [*…*] My routine was literally that I doped myself in the morning to stay awake, train hard and be active. In the evening, I took all kinds of sedatives just to be able to sleep.”*

By enforcing such control, Karel perceived that he “was becoming the body” as his body dominated his life and negative bodily manifestations, paradoxically, gradually extended beyond his control. His attempts to further control his body by even stricter means led to an increasing imbalance between his body and other aspects of his self including relationships, a sense of meaningfulness, and physical and mental well-being. Karel: *“My life became extremely self-centered: I am getting ready for bodybuilding competition, the competition is my first priority and fuck everyone else. I had free time after work to spend with my partner, but we never did anything, and it tortured her. I refused to do anything, I felt exhausted, I enjoyed nothing and I was unable to think about anything other than my body. Once again, my girlfriend and other people around me, including at work, suffered the most. [*…*] There were more problems. Even psychological problems. I literally started having panic attacks. I guess it was because of the combination of all these substances. [*…*] I had some moments that I was sitting for 4 hours at the toilet and refused to get up because I had no energy to get up. That was quite an experience. I guess that if I survived this, I would be hard to kill now (laughter).”*

From his perspective, by discontinuing APEDs and other substances, Karel was striving to regain control and restore balance between his body and the other aspects of his self. In this sense, he described looking for a normal, ordinary life, which he would prefer over an out-of-the-ordinary bodybuilding career. Karel: “*I had one last competition, this time really just for fun, and then I sat down and I guess I finally realized that it maybe doesn’t matter how I look, or that the important people in my life don’t care how I look. If I’m chiseled and pumped or a normal guy who goes to work to make a living. I guess I started reconsidering my life. [*…*] Now I want not to care. Intentionally. Now, when somebody puts ice cream in front of me, I want to eat it without feeling guilty and having to go for a walk [to burn it off]. Reconsider it somehow, have a rest from it all, and when I don’t feel like going to the gym for a week, I just don’t go. Because I don’t feel like it. Why would I go there?!”*

Similar yet different themes emerged in Daniel’s narrative. Daniel also described a pattern of behaviors through which he attempted to enact excessive control of his body, which gradually shifted to a loss of control because he developed an eating disorder. However, Daniel provided different motivation for enacting such control over his body. Throughout his narrative, Daniel described himself as lacking confidence, which resulted in his being excessively sensitive to the expectations of other people and trying to meet such expectations. He even described his beginnings in fitness as being motivated by his perception of himself as untalented. Daniel: *“I needed sports, my body needed them, but I didn’t know what to do because I believed that for any team sport I was too tall, too thin, without coordination, without movement, so what else was I supposed to do? [Fitness] was easy.”* Later, he began to follow an extreme training regimen prescribed by his coaches with the goal of participating in fitness competitions. However, even in this context, he described a lack of confidence and doubts about his sports predispositions, which were further enhanced by his competitive experience. Daniel: *“At the competitions, I was the tallest and also the skinniest. I looked like a spider. I did so much dieting that I lost even the little muscle I had. I was dry, no pump, I was flat. And after competitions, I had the same feeling I had before. That I am not good enough, that there is no future in this; why do it, why bother?”*

Daniel described himself as very sensitive to other people’s expectations; to maintain a sense of self-worth, he focused on developing his body through a strict training regimen and a lifestyle that was significantly different from what he perceived as normal in his peers. Daniel: *“I don’t know anything. I studied at school, which turned out to be useless to me. I couldn’t be a waiter, I don’t know how to cook [or] clean. I cannot change a light bulb. I know absolutely nothing. I couldn’t even change a tire on my bike. I just don’t know anything. So I said to myself that I have to bet everything on one card. My looks!”* As in the case of Karel, Daniel perceived that through such an extreme focus on his appearance, he was “becoming his body” and was paradoxically losing the control he was striving for. The body became a dominant part of his self-concept because he was developing an eating disorder. This process culminated after he moved to the United States where he was offered a job as a model but was considered “too muscular”. Daniel: *“They told me that the face was good, all great, but that I was too muscular and that I was just too big for the top brands such as Versace or Armani. But I became kind of stuck, I saw a chance that it might work out after all. I just wanted to give it a try. So I began fasting. [*…*] I wanted the modeling so bad that I developed a habit of not eating anything for 3 days, I just drank water. [*…*] I was so crazy that I didn’t eat and then I gorged myself like a maniac. My weight regularly changed from 8 to 10 kg during a single week.”*

In this way, he perceived that he “lost himself to his body,” that his striving to become extraordinary in the eyes of other people and begin a successful modeling career severely compromised his overall well-being. Daniel: *“Unhappiness, depression, it all stemmed from the eating disorder, everything. I was unbelievably depressed. I remember that my weekends were that on Friday, I locked myself in my room and did not go out until Monday morning. [*…*] I had absolutely no friends, it was a very difficult period.”* Interestingly, while Karel perceived APEDs and other substances as the reason why he lost control over his body, Daniel experienced his use of APEDs in an opposite way. During the time he experienced such profound loss of control over his body, he began taking testosterone, which he retrospectively considered to be the main factor helping him regain control of his body and “find himself.” Daniel: “*After about 3 weeks [of taking testosterone], I realized that I woke up in the morning and I was happy. I had never had so much energy in my life, how’s that possible? [*…*] And my brain started working at a completely different level. I was surprised and couldn’t recognize myself. I said to myself ‘shit, I like that, I feel like a superman.’ [*…*] That’s why I see it as the best decision of the last 4–5 years. It put my life back on track, I literally feel as if I poured rocket fuel into the engine.”*

Daniel described that he finally freed himself from the expectations of other people and chose what he perceived as an “ordinary career” of a fitness coach. At the same time, that ordinary career facilitated his sense of meaningfulness as he perceived himself as a productive member of the community in which he lived. Later, he seriously considered returning to bodybuilding competitions and trying to become “somebody in the world of fitness”, which would, from his perspective, demand that he starts using other types of APEDs. Daniel: “*Today, you cannot become a fitness personality without steroids. [*…*] The bar is so high that you cannot make it without steroids. You cannot. And I told myself, I want to be one of those fitness people, I want to get an invitation to the Fitness Expo in Dallas. I want to get an invitation to the Fitness Expo in Las Vegas.”* However, he decided against it as he thought that by taking more APEDs, he would return to acting on the expectations of other people, not his own. He saw this as threatening his hard-regained control over his life and compromising the balance between his “extraordinary” and “ordinary” selves. Daniel: “*I realized one thing that helped me. I won’t be a soccer player, I won’t be a model, I won’t be a top fitness celebrity. I am Daniel, I like what I do, and I think that I am quite good at it, I know how to talk to people, they trust me, they like me, so I should use what I have and not try to become somebody I am not. That helped me the most. As soon as I realized this in my head, I became maximally satisfied and happy. But it was a process in which I went through hell, before I got here, to this mental place.”*

#### Robert and Petr: Becoming Experts

While in Karel’s and Daniel’s narratives the dominant themes related to the body and a (lack of) control, the main themes in the narratives of Petr and Robert were quite different. The body, paradoxically, played a relatively minor role in how they described their bodybuilding careers as they emphasized the development of knowledge and experience and striving to “become experts” as the main motive for their use of APEDs. In comparison to Karel’s and Daniel’s narratives, this facilitated an approach in which Petr and Robert were subjectively able to balance both their striving to develop their expertise and their “normal” lives.

While Robert described being extremely skinny during his teenage years, he perceived the development of his body as a byproduct of bodybuilding. He considered enjoyment and psychological self-improvement to be his main motivation to start bodybuilding. Robert: *“I was incredibly skinny. I had 188 cm and about 47 kg, which was borderline malnourishment. So I did not eat, I did not exercise, I was kind of passive. [*…*] I saw [how skinny I was], and people around me saw it, you just could not overlook it. But I definitely did not start exercising because I would be unhappy with the way I looked. I’ve never cared about it too much. Today, of course, I work out to look better. But my physical appearance has never been too important for me. [*…*] I started exercising to have fun, and if it also made me to look better, it would only be a benefit.”*

Throughout his bodybuilding career, Robert described an individualized scientific approach to his training in which he experimented with his body and its reactions to different types of training as well as with himself and his own limits. Robert: *“I said to myself that I am going to go all the way. I wanted to try the hardcore bodybuilding lifestyle in the bulk phase, so every day I had the meals ready, weighed, I ate on time, I functioned just like a pro bodybuilder during the bulk phase, getting ready for competition. I said to myself that I just want to try how it’s going to be, if I can make it. I needed to find out whether I could handle it because if I couldn’t, there would be no point to continue.”* In this manner, he also described his motivation to use APEDs – not as a way to develop his body but as a way to extend his knowledge. Robert: *“I kind of approached [using APEDs] as an interest of mine in which I aimed to find out as much as I could and improve myself in the using. It sounds a bit absurd but I mean improve myself in terms of getting as much information as possible.* […] *So I got interested in learning about how [the APEDs] work, what kind of effects on humans they have and so on, rather than saying, alright, I will be a bodybuilder, let’s dope. Or that I have to dope to look better. I got interested in that stuff as a concept, conceptually.”*

Throughout his APED experience, Robert described implementing this type of scientific approach whereby he focused on experimenting with the effects APEDs on himself and his body and on finding the most efficient ways to use APEDs. For example, he went through six cycles of APEDs, and he described that in each cycle, he specifically focused on experimenting with different types of APEDs or with the effects of the APEDs in the context of different types of training. In each cycle, he also described learning specific things, such as how he responded to specific steroids, developing knowledge that he tried to integrate into his own framework of APED use. Robert: *“Regarding the substances, I always wanted to try something new. In the second cycle, I aimed to gain weight. In the third cycle, I wanted to try diet, caloric deficit, make changes to my training and try different substances to learn what effects they have on the weight cut and the diet. [*…*] I started reading medical studies. So I got deeper into it than a large majority of the people who use these substances. And the sixth cycle was focused on testing all these principles to prove whether they work or not and find some shortcuts, a new method that would move it to a new level, not necessarily in [terms of] effectiveness, so I would grow even more, but to get positive effects with absolutely minimal amounts of these substances or to avoid the negative effects. I must say that this turned out great, I verified the effectiveness of many of these principles. In some, of course, I found out that they don’t work, that’s how it is.”*

At the time of the interview, Robert was going through a phase in life during which he moved out of his parents’ house and began living with his girlfriend, a competitor in bodyfitness. At the time of the interview, he was not using APEDs. He did not experience any negative effects, but he believed he did not train at a level at which APEDs would be beneficial, as he focused on his work and the competitive career of his girlfriend. He did not exclude that he would continue experimenting with the effects of APEDs in the future, but he did not consider it of primary importance. Robert: *“For the first time in several years, I feel that I don’t know [whether I want to continue experimenting with APEDs]. It’s because I experienced a big change in my life as I moved out from my parents’ house because I found a girlfriend and we moved together to a new flat. So my life changed a lot, I have more responsibility, I find my work more important to make money, to take care of ourselves and so on. My girlfriend also competes in bodyfitness and I said to myself that I am fine with that, that our primary focus in trainings and nutrition will be on her and I will just trot along. Now I don’t find it so important, or I consider other things to be more important.”*

In a similar way, Petr’s sense making about APEDs occurred in the context of developing his knowledge of bodybuilding and acquiring experience that would be useful in his coaching career rather than using APEDs to develop his body. Petr only had one experience with APEDs in which he also employed a scientific approach in a preparation phase as well as in the reflections on the APED experience. Petr: *“It was just everywhere, everybody was taking it, but, specifically for me, it wasn’t why I did it. I wanted to experience what it’s like. We often talked about it, listened about it, we saw what it did to people, and stuff like that. I knew I wanted to follow a career in which I would train people, passing on information that I have collected. So I wanted to know how it is in real life. I knew I probably wouldn’t participate in competitions, but at least I wanted to try it, so I would know what I was talking about, how it is in reality. And it met these expectations. Prior [to taking APEDs], there were months of reading articles, collecting information, [learning about the] experiences of other people, what would happen when this, what to do when that, we did not do it out of the blue. But there were moments that surprised me, which you wouldn’t find in the books. So it gave me what I wanted from it.”*

While Petr described benefits for his training, he also experienced negative effects that affected his psychological well-being as well as his relationships. Petr: *“We could endure more than before. Suddenly, our workout sessions did not last an hour or an hour and 5 min but rather an hour and a half, and it felt like nothing. [*…*] As for negative [effects], they were mostly about my mental state. Around halfway into the chemical part of post-cycle therapy, it hit me really hard because this shit is even worse than steroids. Halfway through this part I was like a menopausal woman. [*…*] People would say something to me, and I was all-melancholic about it, I took everything personally, it was absolutely awful. [*…*] I remember a situation when my girlfriend was getting out of the car and the door just slipped out of her hand, it closed maybe 1% harder than it normally would. I started yelling, screaming, the poor girl almost cried. Just horrible. She really suffered at that time.”* Because of these negative effects, he decided not to repeat the experience. This decision was subjectively made easier because he had experimented with APEDs primarily to develop knowledge that would be useful in his coaching career rather than in training his body. He did not entirely exclude that he would use APEDs in the future but only if it would not interfere with other areas of his life such as having a stable relationship and raising a family.

#### Martin and Denis: Becoming a Competitor vs. Enjoying Life

Of all participants, Martin and Denis reported the most extensive participation in bodybuilding competitions and went through the highest number of APED cycles. At the same time, they made sense of their bodybuilding careers, competitive experience and APED use in contrasting ways. Martin was the only participant who tied his APED experience directly to participation in bodybuilding competitions. He described that his early motivation to begin bodybuilding was that he was “fat,” and he aimed to gain muscle to improve his looks. Martin: *“It motivated me that I was fat since I was a child, not extremely but I weighed around 100 kilos. So I dropped to 70 and kind of started training. [*…*] [I wanted to] look better, gain muscle. You know [I thought that I would] come to the gym and put on 10 kilos of muscle in a year. It wasn’t like that.”* He was disappointed by his slow progress toward his bodybuilding goals, which he attributed to inexperience and bad habits from his life before sports. Martin: *“At first, I thought I was doing well. There was no doping and stuff like that back then. But I did not see much progress. The body seemed to respond at first, but soon I stopped seeing any improvement. [*…*] I wasn’t cutting corners in training and eating, although looking back I can see that I did it wrong. The trainings were not intensive enough, and there was no periodization. And on top of that, I did not eat enough. Not that I would eat wrong but as I used to be fat, I was not used to having the caloric intake at such a high level.”*

Martin perceived his first bodybuilding competitions as a significant step-up in his bodybuilding career. He saw his participation in bodybuilding competitions as enhancing a sense of meaningfulness in his life because they provided a long-term aim and structure to his efforts. Martin: “*My goal and success, since I had always been a fat child, was just to prepare for competition. [*…*] I want to have that goal, I want to be preparing for the competition and I want my day structured in this way that I enjoy. Not that I [feel that I] have to prepare for the competition. I don’t have to. When the competition comes, I am totally bummed. But I enjoy the year-round process that leads you somewhere. I used to have a good job, good money, but I didn’t want to focus only on work, I wanted something like this. [*…*] I always had some long-term goal. I wasn’t lost in my life. I did not come [home] at four from work and [think] what should I do, so let’s have a beer and watch TV. Even when I watched some TV, I had a good feeling that I ate the food I was supposed to, I had my training, I was in good shape.”* In this way, Martin described his bodybuilding career as a progression of bodybuilding competitions, which he inevitably linked to using APEDs. Martin: *The last preparation was a lot more professional, I was able to train better, eat better, and with regard to the substances, it was kind of the same as before. You cycle the ten basic substances round and round. [*…*] Testosterone ethanate, nandrolone in the same amount, maybe 10–20% more, methandienone on top of that. And in the cutting phase there was quite a lot of stuff, fast testosterone, drostanolone, I had also trenbolone, which is quite a strong injected substance. [For] oral steroids, I had stanozolol, and oxandrolone.”*

At the time of the interview, Martin had stopped competing and using APEDs because he planned to start a family with his girlfriend. They had not been able to conceive so far, which he partially attributed to his previous use of APEDs. Martin maintained a rigorous training regimen that he perceived as important for his psychological well-being and a sense of meaningfulness. As he positively reflected on his APED experience, he did not exclude that he would return to competing and using APEDs after he had children. Martin: *“It’s like driving a Trabant and then getting into a Ferrari. [*…*] I am not entirely sure now that I want to compete at any cost, but if we manage to conceive and if I have the capacity to prepare for a competition without hurting my work and other things, then I might compete again.”*

While Denis also participated in several bodybuilding competitions, he described his competitive motivation differently than Martin. Martin participated in competitions because they gave him a sense of direction and meaning in life, and he approached APEDs as a mandatory part of the process. Denis described his participation in competitions as a negative experience with extensive demands that significantly affected his psychological well-being as well as other domains of life such as relationships and career. Denis: *“The international competition] was quite unpleasant. It was terrible, in fact. I think, when you try it, so anybody with any sense, in their right mind, would tell you that for the 5 min at the podium, it is not worth it at all. Looking back, I don’t understand [how] somebody [could pursue] this competitive career and enjoy it. Getting ready for so long. I know a guy who was preparing for a competition, and he ended up on dialysis twice. On dialysis! And even then he went to the competition. All for the 5 min at the podium. I just don’t get it.”* After such negative experiences, Denis decided to quit competing and focus on enjoyment he was getting from bodybuilding with an explicit aim to develop his body as much as possible. Denis: *“[After the last competition] it was pretty wild. I was clean for some time because I was on the stuff for a year. And then I started bulking up because I wanted to catch up with what I lost. I did not want to prepare for competitions anymore, but I wanted to become the biggest freak there was. And fuck all competitions, I was pretty clear about that.”*

Contrary to Martin, Denis did not see using APEDs as intertwined with bodybuilding competitions, but he described it as a way to increase his enjoyment both in bodybuilding and in life. He described his first APEDs cycle as a somewhat impulsive decision based on information found on the internet and motivated by the fact that he wanted to extend his bodily limits. Denis: “*I weighed 95 kilos at the time, my performance was quite decent, I had [achieved] some records in the gym, but I thought it was going slow, that it really sucked, actually. Because I wanted to get over 100 kilos but it just wouldn’t move. And I wasn’t satisfied at all with the shape I was in, I looked like shit. So I thought that I would try [APEDs]. And it blasted me to a completely different level.”* Reflecting on his APED experience, Denis perceived it not only as improving his body but also as increasing his enjoyment of training, self-confidence and overall wellbeing, which transferred to his life beyond sports, especially to his work. Denis: *“It is like you were driving the lowest model of Fabia and then sat in a new BMW. It is something completely different because you don’t feel this way only during the training, in which you, by the way, feel incredible, but you feel like that all day. All the time. Because, on top of everything, you have higher self-confidence, better appetite, higher sex drive, all these things. You feel better all the time. It improves the quality of your life.”*

Within this mindset, Denis described a more reckless approach to APEDs than the other participants did. As he experienced APEDs as beneficial to his bodybuilding goals as well as to his life outside sports, he appeared to rationalize the negative effects of APEDs to allow himself to feel comfortable using them continuously. For example, he experienced a possibly serious health condition that he attributed to his excessive use of APEDs; however, he described being largely undeterred in his intention to continue using APEDs. Denis*: “It was quite interesting, what happened to me recently, during the last bulking phase. I started with growth hormone, I bought growth hormone, and I was taking about three units. It was my last cycle, so I stacked it quite a bit. I started with 900 mg of testosterone, then I took 600 mg of nandrolone, and some 800 mg of boldenone, which is a lot! I added 5 units of growth hormone into the mix, and then I added some 50–100 mg of methandienone a day. Anybody would tell you that it was quite a stack. It was great! It was absolutely awesome. Until I got a feeling that I started pissing blood. And that was bad. I got scared shitless that something bad was happening, that I fucked up. [*…*] After that I stopped using, I did a short detox. And I got tested, and it was just like before. No problem at all because the liver has good regeneration ability. It is completely miraculous in this way.”*

While Denis recognized that there may be a clash between his bodybuilding career and other life domains in the future, he currently continued using APEDs and was dedicated to pursuing his high achievement goals, which was in his view facilitated by the use of APEDs. Denis: “*Now I approach [the use of APEDs] that I am in a life phase, when I am single, and I can live this selfish life and completely focus on my training. When somebody appears in the future, family or something like that, it is obvious that I will have to put these things on the backburner. I learned this already, so it is kind of my hobby for now.”*

### Different Contexts of APED Sense Making: Unwanted, Extraordinary, and Ordinary Selves

To summarize all the individual cases, the participants made sense of APEDs in the context of their self-concepts, including self-schemas of who they are, who they had been, and who they could be and wanted to become, both within and outside bodybuilding. In this way, they perceived APEDs not exclusively as an instrument to enhance their bodies but as a tool they used to “enhance their selves” as they generally referred to APEDs in the context of striving to become extraordinary individuals or to lead out-of-the-ordinary lives. At the same time, they balanced these “extraordinary selves” with other aspects of their identities including relationships, professional careers, and general health and well-being. They made sense of their experiences with APEDs in the context of both of these selves, which included their striving to become extraordinary through their bodies and to lead ordinary, balanced lives. The ways in which the use of APEDs contributed to both of these constructions appeared to determine the participants’ views on continuing or discontinuing APED use.

For example, to compare two contrasting cases, Daniel made sense of his use of testosterone as a key factor that allowed him to regain control in his life, develop overall well-being, and find a life purpose, whereas Karel perceived his APED experience as leading to loss of control, feelings of meaninglessness, the breakdown of relationships, and general ill-being. Daniel: *I think that [using testosterone] was one of the best decisions I could make in my life. It allowed me to work again, to have a vision, to follow my dreams. Before, when I was so depressed, I was glad it was evening, morning, evening, morning, my life was shit. So, taking testosterone is something really important for me, I feel incredible, I don’t want to stop taking it, I am not willing to stop and I will not stop.”* Karel: *“Unfortunately, I’d say that the main reason for stopping [using APEDs] was how horribly I felt both physically and mentally. It is only now, after some time has passed, that I am starting to realize how badly I treated other people in my worst moments. At that time, I often told myself that I liked being on my own, that I was happy and so on, but now I wonder whether I really want to spend my life alone only because I want to look good. I am not saying it’s wrong. It does not have to be wrong. I just wonder whether it is for me and whether I am willing to accept it.”*

As we have shown in the section above, the participants construed themselves through narratives of their bodybuilding careers and APED experiences in different ways. However, they all structured these narratives through several superordinate themes. These themes, which we labeled as “unwanted self,” “extraordinary self,” and “ordinary self,” were approximately related to different stages of their bodybuilding careers. First, in the context of their beginnings, the participants talked, both explicitly and implicitly, about their “unwanted selves,” i.e., about what they did not like about themselves at that time and how it determined what they strived to become or, vice versa, un-become through their engagement with bodybuilding. In these constructions of unwanted selves, the participants included unattractive bodies but, perhaps more importantly, a lack of agency in their lives. They related such a lack of agency to inexperience, insufficient knowledge, low confidence, a lack of self-regulation abilities and bodily limits as well as the need to fulfill the expectations of other people. However, the participants viewed APEDs as insufficient to help them move from these unwanted selves at this point, even using similar phrases to describe misplaced APED use in the early context. Karel: *“*I *expected miracles, but the thing is that if you are a shit and you take AAS, you just become a bigger shit. If you look naturally good even without any substances, if you have trained with a good coach for years and then you take something to get ready for a competition or you are just curious and give it a try, then the difference is huge.”* Martin: *“No offense, but if you look like shit, you should not think about taking steroids.”*

The participants perceived APEDs not as instruments that should be used to improve deficiencies that could be mitigated in other ways but rather as an instrument to facilitate their self-actualization and development beyond the ordinary. In this context, they developed a theme of “becoming” in which they described a striving to actualize their potential and, in a sense, to “become extraordinary,” not necessarily as bodybuilders but as people. Therefore, they perceived such becoming taking place through bodybuilding rather than in the bodybuilding alone. While their sports ambitions did not surpass the amateur level or they were not interested in bodybuilding competitions at all, they strived to develop bodies, personal characteristics, knowledge, experiences, or social standing that exceeded what they perceived as “normal” or “ordinary” in the general population. In this context, they made sense of APEDs as a factor that facilitated or was even necessary for such becoming.

Denis: *“I had no self-confidence, and [using APEDs] helped me incredibly to start believing in myself. And a lot of things originated from that, even that I have my own business and was able to make myself go for it. It really helped. Even to become self-sufficient, in these difficult situations that happen in life, when you screw up and now you know that you are in a tight spot and need to do something about it. It will help you dig in and say to yourself, look I cannot leave it like that, I am going to do something about it. So from this perspective, I have to see it extremely positively. Personally, I don’t believe that I would be where I am now without [APEDs].”* Robert: *“[If it] weren’t for these substances, I don’t think that I would [have gone] so deep into it. Having life so well structured or developing such knowledge. That I learned the discipline and went through the necessary training, cooking, preparing meals, time management: these things helped me also in my personal and work life. So I think [APEDs] gave me more than they took away.”*

In the third superordinate theme, the participants referred to “balancing” or “losing” different aspects of the self, which emerged as a consequence of their striving to become extraordinary. In this way, the participants referred to an impact of such becoming on other aspects of the self, which they described in terms of a need to “be normal” or to lead balanced, ordinary lives. All participants described some ways in which their striving to become extraordinary through bodybuilding and using APEDs negatively affected their health, physical and mental well-being, work life or relationships. The tension between these two selves – “ordinary” and “extraordinary” – determined the ways in which they made sense of APEDs and their continuing use. In some cases, they described such impacts as “balancing”, which demanded relatively minor adjustments, either because the negative effects were not severe or because the becoming demanded relatively minor adjustments. In other cases, the participants described these negative impacts predominantly in terms of “losing” as they perceived that they had to make large changes in the ways in which they approached their bodybuilding careers and use of APEDs that they considered a key factor of such losing.

Karel: *“Right now, I feel so much disgust that I’d say that I cannot imagine ever going back to this stuff and focusing only on my body and how my body looks. But never say never. And I know that I already said the sentence, “I never want to do this again” before and did not stick to my words. So to be completely honest, I don’t know. The only thing I do know is that while preparing for the last competition, I tried to do without [APEDs], just for fun really, I could do it without any sleeping pills, without any drugs and stuff like that. So it can be done without it. It can be done without chemicals.”*

## Discussion and Conclusion

In the previous section, we analyzed the ways in which amateur bodybuilders made sense of their experiences with APEDs in the context of their bodybuilding careers and other life domains. We argue that the participants’ decision to use and stop using APEDs needs to be understood from a broader perspective of how they perceive their lives inside and outside the context of sports, which emerged from the analysis as a tension between their “extraordinary” and “ordinary” selves. In other words, the participants made sense of their APED use in the contexts of their striving to “become extraordinary” through sports and, at the same time, to remain normal or ordinary in other life domains. At the same time, these general themes showed wide variability among our participants as they related to more specific themes of body and (a loss of) control in Karel and Daniel, knowledge, experience and expertise in Robert and Petr, competition and a sense of meaning in Martin, or enjoyment and quality of life in Denis.

We implemented a phenomenological perspective (e.g., Smith and Osborn, 2007; Shinebourne and Smith, 2009; Smith, 2019) and the concept of the self (e.g., Markus and Nurius, 1986; Markus and Wurf, 1987; Kihlstrom and Kihlstrom, 1999; Andreasson and Johansson, 2020) as integrating frameworks in our analysis. In this way, we strived to complement the existing research on the use of APEDs by taking a relatively neglected perspective emphasizing individual subjective experience over approaches that provide generalized objective accounts of APED use. Such in-depth insights illustrate the complexity and multiplicity of the motives for the use of APEDs, which cannot be reduced solely to a limited set of variables such as sports motivation (Barkoukis et al., 2011, 2013; Hodge et al., 2013; Mudrak et al., 2018), ego achievement goal orientation (Sas-Nowosielski, 2006; Sas-Nowosielski and Swiatkowska, 2008) or doping-related attitudes (Chan et al., 2015). In relation to bodybuilding and the use of APEDs, the participants described intrinsic and extrinsic motives, a competition orientation and a focus on self-development, or positive and negative attitudes toward APEDs. Based on our analysis, we argue that an understanding of why athletes decide to use APEDs may be obtained by considering their self-concepts as consisting of multiple selves (Markus and Nurius, 1986; Markus and Wurf, 1987) that activate different types of sense making about APEDs, some of which may facilitate the use of APEDs, while others may prevent it.

To understand the multiplicity of motives for the use of APEDs, we find very useful the typology of APED users in fitness and strength training environments introduced by Christiansen et al. (2017) and Christiansen and Flegal (2020), which in many ways corresponds to our findings. Christiansen et al. (2017) argue that APED users differ in two dimensions including their focus on the effectiveness of APEDs and their acceptance of APED-related risk. When applying this typology to our participants, Robert fell into the high effectiveness/low risk-oriented expert type as he implemented a scientific approach in finding the best possible effects of APEDs while striving to eliminate their possible negative side effects. In comparison, Denis embodied the YOLO type as he described high acceptance of risk, a relatively *ad hoc* approach to the use of APEDs and high seeking of excitement. In a similar manner, we could classify Daniel into the well-being type, as he emphasized the positive effects of relatively limited use of testosterone on his overall well-being, or Martin into the athlete type, as he related his APED use almost exclusively to participation in competitions. At the same time, based on our results, we argue that using such a typology entails significant limitations as our participants often described their APED use in ways that corresponded to multiple types. This was even more obvious when we considered different phases of our participants’ bodybuilding careers, as they would correspond to different types at different stages of their careers. The second limitation of applying such a typology may be that it somewhat neglects life domains outside bodybuilding, which may have, as our results and other studies (Erickson et al., 2015) illustrate, a central role in the participants’ sense making about APEDs.

Our results emphasizing the participants’ subjective experience also provide a contrast to objective, pathologizing views of bodybuilding and the use of APEDs. These approaches come from medical and social constructivist perspectives (e.g., Klein, 1993; Pope et al., 1993; Wiegers, 1998; Davis and Scott-Robertson, 2000; Peters and Phelps, 2001; Goldfield et al., 2007; Skemp et al., 2013) and describe the use of APEDs as resulting from insecurity, vulnerability and a gender-related sense of inadequacy. While these approaches undoubtedly provide relevant explanations for why people use APEDs in the bodybuilding context, they appear to be somewhat disconnected from the subjective experience of actual APED users. In our research, the participants generally did not perceive APEDs as a means of fixing deficiencies but rather as a path toward self-actualization. In this way, they used APEDs not only to develop their bodies but also to enhance their sense of meaning, well-being and quality of life and to gain knowledge and expertise. In fact, Karel was the only participant who explicitly described a trajectory of APED use that he, himself, considered pathological as he strived to develop his body through excessive self-control, which shifted into a loss of control subjectively perceived as a “loss of self.” Other participants generally perceived their APED use in a way in which the positive experiences overweighed the negative experiences. At the same time, they tied their APED use to specific periods of their life in which the benefits could be fully utilized while they perceived the use of APEDs in other life stages more negatively.

Based on the statements of our participants, we find their self-concepts to be anchored in their bodies and manifestations of their bodies and socially embedded in various social contexts, representing an intersection between the bodily and social aspects of human experience (Shilling, 2012). As shown by Brown (1999), in the bodybuilding context, the body represents a “hyperreflexive physical project” and, at the same time, a “place of social communication” (Brown, 1999, p. 84) where athletes signal their masculinity but are simultaneously forced to confront the fact that their body and lifestyle do not align with what the general society considers to be normal. From their statements, it is clear that our participants had to deal with dilemmas stemming from their extreme focus on their own bodies and the simultaneous need to build positive relationships with other people. Some of our participants perceived their experience with APEDs as a behavior that seriously damaged the balance between these two poles, and they strove to develop a balanced self-concept that would encompass both poles. Interestingly, for some of our participants, relative success in finding this balance supported a sense making that created a possibility of future APED use.

Many studies have focused on analyzing and explaining social processes that drive the transformation of a regular gym-goer to an APED user (Monaghan, 2001a; Brissonneau and Montez De Oca, 2018; Coquet et al., 2018; Andreasson and Johansson, 2019). Our participants considered an evolution of their bodybuilding careers beyond the stage including the use of APEDs. They considered returning to APED use on the condition that they would be able to balance the demands of their multiple selves, including different life domains, which they mostly believed possible. Our findings thus help explain the persistence of APEDs and add to previous findings based on qualitative studies of bodybuilding and risk practices associated with this sport (Klein, 1993; Monaghan, 2001a; Brissonneau and Montez De Oca, 2018; Coquet et al., 2018). Monaghan (2002) claims that the use of steroids has been rationalized within the bodybuilding context as a legitimate means to an end; negative perspectives are rejected by “condemning the condemners,” and steroids are claimed to have little serious health effects and no negative social consequences. However, as we find in the descriptions of our participants, bodybuilders may change such rationalizing sense making in the course of their bodybuilding careers, depending on the salience of their different selves during specific developmental periods. At the start of their bodybuilding career, our participants described going through a period of misguided experimentation with APEDs while they saw APEDs as an indispensable step to achieving goals stemming from their “extraordinary self.” At the end of their career, they perceived APEDs as an obstacle to reaching goals stemming from their “ordinary self.” In this way, the meaning that the bodybuilders assigned to their APED experiences changed during the course of their careers; consideration of their self-concepts may provide an explanation for such changes and a means of identifying “exit points” providing opportunities for change (Petróczi and Aidman, 2008).

We need to consider the limitations of our study. The first limitation stems from the “double sense making” present in our methodological approach. This means that the descriptions of our participants represent subjective interpretations of their experience and should be approached as such. Furthermore, our analysis of the categories, themes and interpretations of the participants’ sense making, while supported by other research, is based on our subjective decisions, and other researchers could approach the same data through different themes or theoretical frameworks. While our analysis provided insights into the motives of amateur bodybuilders to use and stop using APEDs, this kind of analysis provided no information about the prevalence of these themes. Additionally, as the research focused on a sensitive topic with possibly harmful consequences for the participants, they might not have been entirely honest in their answers. Finally, the study was carried out in the national context of the Czech Republic with specific APED-related policies, which provided different frameworks for the sense-making of our participants than other national contexts, such as Scandinavian countries (e.g., Andreasson and Johansson, 2020), in which APED use is illegal and may have potential negative legal consequences for users of APEDs.

As far as it is possible to draw any general conclusions from the above findings, we may say that our study highlights the complexity of the subjective reasons for the use of APEDs and the role of multiple selves in bodybuilders’ sense making about APEDs. We showed that amateur bodybuilders made sense of their APED use in different ways that were related not only to their body or competitive success but also to other psychological outcomes, such as agency, sense of meaning, self-development and quality of life, which the participants included in their “extraordinary selves.” In this way, we may argue that these bodybuilders used APEDs not only to enhance their appearance or performance but, maybe more importantly, to enhance their selves. Within such sense making, a potential “exit point” emerged from a tension between the “extraordinary self” and “ordinary self,” i.e., our participants thought about stopping using APEDs predominantly in the context of losing in subjectively important domains, such as subjective well-being, relationships or work, as they constructed what they considered a normal, ordinary life.

## Data Availability Statement

The original contributions generated for this study are included in the article/supplementary material, further inquiries can be directed to the corresponding authors.

## Ethics Statement

The studies involving human participants were reviewed and approved by Ethical Commission of the Ethics Committee of the Faculty of Physical Education and Sport of Charles University. The patients/participants provided their written informed consent to participate in this study.

## Author Contributions

JuM: conceptualization, data curation, formal analysis, investigation, methodology, and writing – original draft. JiM: conceptualization, data curation, formal analysis, funding acquisition, investigation, methodology, visualization, and writing – original draft. PS: conceptualization, data curation, formal analysis, funding acquisition, investigation, methodology, supervision, and writing – original draft. All authors contributed to the article and approved the submitted version.

## Conflict of Interest

The authors declare that the research was conducted in the absence of any commercial or financial relationships that could be construed as a potential conflict of interest.
